# Total and regional body adiposity increases during menopause—evidence from a follow‐up study

**DOI:** 10.1111/acel.13621

**Published:** 2022-05-04

**Authors:** Hanna‐Kaarina Juppi, Sarianna Sipilä, Vasco Fachada, Matti Hyvärinen, Neil Cronin, Pauliina Aukee, Jari E. Karppinen, Harri Selänne, Urho M. Kujala, Vuokko Kovanen, Sira Karvinen, Eija K. Laakkonen

**Affiliations:** ^1^ 4168 Gerontology Research Center and Faculty of Sport and Health Sciences University of Jyväskylä Jyväskylä Finland; ^2^ 4168 Faculty of Sport and Health Sciences University of Jyväskylä Jyväskylä Finland; ^3^ Neuromuscular Research Center, Faculty of Sport and Health Sciences University of Jyväskylä Jyväskylä Finland; ^4^ 2376 School of Sport and Exercise University of Gloucestershire Cheltenham UK; ^5^ Department of Obstetrics and Gynecology Central Finland Health Care District Jyväskylä Finland; ^6^ 4168 Department of Psychology University of Jyväskylä Jyväskylä Finland

**Keywords:** adipokine, body fat distribution, longitudinal studies, obesity, perimenopause, physical activity

## Abstract

For women, menopausal transition is a time of significant hormonal changes, which may contribute to altered body composition and regional adipose tissue accumulation. Excess adiposity, and especially adipose tissue accumulation in the central body region, increases women's risk of cardiovascular and metabolic conditions and affects physical functioning. We investigated the associations between menopausal progression and total and regional body adiposity measured with dual‐energy X‐ray absorptiometry and computed tomography in two longitudinal cohort studies of women aged 47–55 (*n* = 230 and 148, mean follow‐up times 1.3 ± 0.7 and 3.9 ± 0.2 years, mean baseline BMI 25.5 kg/m^2^). We also examined associations between menopausal progression and skeletal muscle fiber characteristics, as well as adipose tissue‐derived adipokines. Relative increases of 2%–14% were observed in regional and total body adiposity measures, with a pronounced fat mass increase in the android area (4% and 14% during short‐ and long‐term follow‐ups). Muscle fiber oxidative and glycolytic capacities and intracellular adiposity were not affected by menopause, but were differentially correlated with total and regional body adiposity at different menopausal stages. Menopausal progression and regional adipose tissue masses were positively associated with serum adiponectin and leptin, and negatively associated with resistin levels. Higher diet quality and physical activity level were also inversely associated with several body adiposity measures. Therefore, healthy lifestyle habits before and during menopause might delay the onset of severe metabolic conditions in women.

## Graphical abstract

Menopause is associated with increases in total and regional body adiposity, with a pronounced increase in the android area. Adipose tissue‐derived adipokines, adiponectin, leptin and resistin, were uniquely associated with menopausal transition. Lifestyle habits, such as higher diet quality and physical activity level, but not external hormone use, were associated with lower adiposity in several body regions.

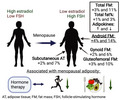

**Abbreviations**
ACC‐PA: Accelerometer‐measured physical activity
BMI: Body mass index
CT: Computed tomography
DHEAS: Sulfated dehydroepiandrosterone
DQS: Diet quality score
DXA: Dual‐energy X‐ray absorptiometry
ERMA: Estrogenic Regulation of Muscle Apoptosis ‐ study
EsmiRs: Estrogen, MicroRNAs and the Risk of Metabolic Dysfunction ‐ study
E2: Estradiol
FM: Fat mass
FSH: Follicle‐stimulating hormone
GPD: α‐glycerophosphate dehydrogenase
HU: Hounsfield units
HT: Hormone therapy
IMCL: Intramyocellular lipid droplet
IUD: Intra‐uterine device
LPL: Lipoprotein lipase
MET: Metabolic equivalent of a task
MVPA: Moderate‐to‐vigorous physical activity
SDH: Succinate dehydrogenase
SHBG: Sex‐hormone binding globulin
SR‐PA: Self‐reported physical activity

## 1 INTRODUCTION

Adipose tissue has an important role in energy storage and hormonal supply (Chait & den Hartigh, 2020). Genetic and environmental factors determine adipose tissue mass and distribution by modulating energy balance and lipid‐related enzyme activity. Subcutaneous adipose tissue serves as a long‐term lipid storage, while visceral adipose tissue is metabolically more active and acts as an acute‐response supplier of systemic fatty acids (Chait & den Hartigh, 2020). Both the mass and the location of adipose tissue are important at the systemic level, as higher gluteofemoral adipose tissue mass has been linked with a better metabolic profile and higher insulin sensitivity, while increased waist adiposity may increase cardiovascular and metabolic risks (Manolopoulos et al., 2010; Peppa et al., 2013). Sex chromosomes and hormones are known to be important mediators of adipose tissue distribution. For example, regional expression of lipoprotein lipase (LPL), the enzyme responsible for releasing fatty acids for storage lipid droplets, differs between the sexes, contributing to the waist‐centered adipose tissue accumulation pattern in men and pear‐like (legs and gluteal area) shape in women (Wang & Eckel, 2009; Wells, 2007). Moreover, sex hormones such as estradiol (E2) and testosterone regulate the expansion and metabolism of adipocytes, and consequently also depot insulin sensitivity (Newell‐Fugate, 2017).

In addition to supplying energy, adipose tissue secretes hormones known as adipokines. Adiponectin, leptin, and resistin are some of the most studied adipokines, and their roles in adipose tissue originated signaling and as indicators of metabolic health have recently been revealed (Recinella et al., 2020). Adiponectin has been shown to act as an insulin sensitizer and is inversely associated with obesity, type 2 diabetes, and metabolic syndrome (Fasshauer & Blüher, 2015). The gluteofemoral adipose depot is a possible source of adiponectin, while lower adiponectin levels are associated with increased visceral adipose tissue (Manolopoulos et al., 2010). Leptin has a role in signaling full energy stores and decreasing appetite, and leptin concentration correlates positively, similar to resistin levels, with insulin resistance and body adiposity (Recinella et al., 2020). Besides metabolic associations, excess adiposity also affects functional capacity. Lower muscle density, reflecting increased adipose tissue infiltration into the muscle compartment, is associated with whole‐body adiposity (Goodpaster et al., 2000) and can lead to decreased muscle power and postural balance (Straight et al., 2019).

At the cellular level, intramyocellular lipid droplets (IMCL) have an important role in energy storage during physical activity. Both IMCL concentration and the metabolic capacity of different muscle fiber types are associated with obesity. For example, increased lipid accumulation in muscle fibers, lower oxidative capacity, and a decreased proportion of type I fibers have all been found to positively correlate with overall adiposity and insulin resistance (He et al., 2001; Tanner et al., 2002). Type I muscle fibers are typically rich in lipid droplets and possess high oxidative capacity, while type II fibers contain fewer lipid droplets and have a higher glycolytic capacity (Schiaffino & Reggiani, 2011). Sex also contributes to differences in lipid droplet metabolism, as women typically have more IMCL compared with men of the same body mass index, yet remain more insulin sensitive (Goossens et al., 2021).

Aging is a major contributor to adipose tissue accumulation, but in women, this seems to accelerate during menopause. Yet, due to a concomitant loss of lean mass (Juppi et al., 2020), weight does not necessarily increase (Greendale et al., 2019). Menopause is characterized by a cessation of ovarian function, which results in low E2 levels and high follicle‐stimulating hormone (FSH) levels. E2 has been suggested to regulate LPL (Wang & Eckel, 2009), while FSH has been linked to the promotion of lipid biosynthesis and is positively associated with leptin and negatively with adiponectin levels in cellular and animal models (Liu et al., 2015). Studies investigating the roles of aging and menopause in increasing adiposity have reported contradictory results. A recent meta‐analysis suggests that aging is the main contributor to increased overall adiposity, while menopause contributes to adipose tissue accumulation in the waist area (Ambikairajah et al., 2019). Although women of reproductive age are more protected from cardiovascular conditions compared with men, menopause seems to remove this advantage concomitantly with the change in adipose tissue distribution. Studies of postmenopausal women using hormone replacement therapy (HT) highlight the beneficial role of female sex hormones, which are associated with lower visceral adipose tissue mass (Papadakis et al., 2018). In addition to hormones, better diet quality and regular physical activity are widely recognized contributors to fat mass and metabolic health management in midlife.

This study examined the longitudinal associations between menopausal transition and the accumulation of total and regional body adiposity, as well as changes in systemic and muscle tissue adiposity markers. We also investigated other potential contributors to the body adiposity changes, including physical activity, diet quality, and the use of external hormones. We hypothesized that menopausal transition would be positively associated with several body adiposity variables from whole body to cellular level, and that the highest relative increase in adiposity would occur in the android region. We also hypothesized that increases in leptin and resistin levels and a decrease in adiponectin levels would occur during menopausal transition.

## 2 RESULTS

### 2.1 Summary statistics and baseline characteristics

**FIGURE 1:**
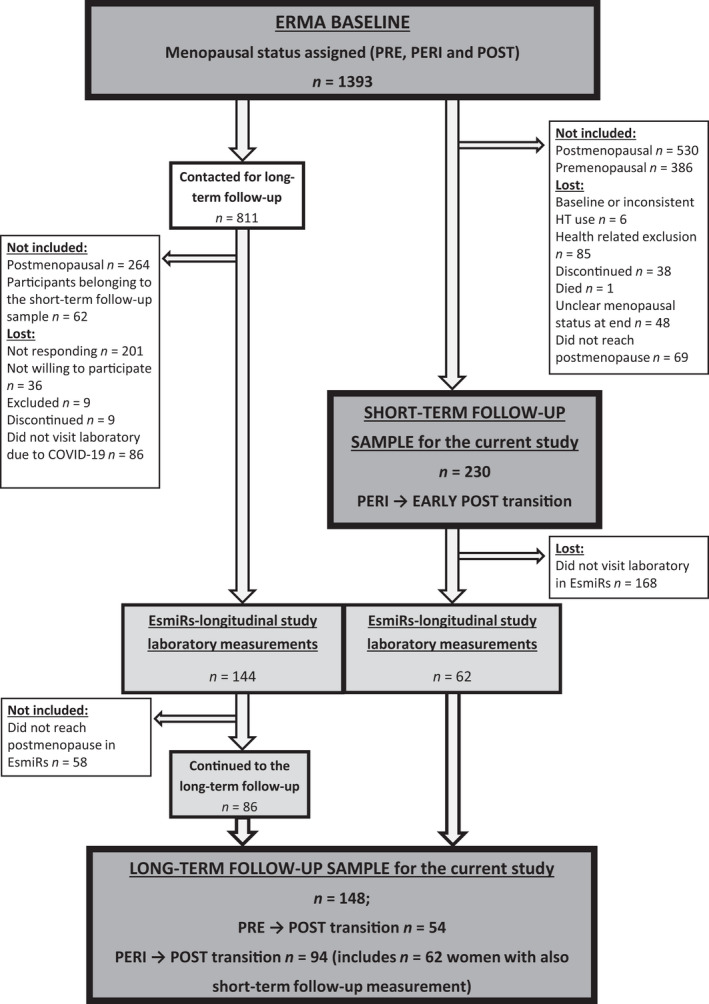
Flow chart of the short‐term (*n* = 230) and long‐term follow‐up studies (*n* = 148). ERMA, Estrogenic Regulation of Muscle Apoptosis; EsmiRs, the Estrogen, MicroRNAs and the Risk of Metabolic Dysfunction; HT, hormone therapy; PRE, premenopausal; PERI, perimenopausal; POST, postmenopausal

The data used were from the Estrogenic Regulation of Muscle Apoptosis (ERMA) (Kovanen et al., 2018) and the Estrogen, MicroRNAs, and the Risk of Metabolic Dysfunction (EsmiRs) (Hyvärinen et al., 2021) studies (Figure 1). All participants were Caucasian women. To be included in the current study, a participant needed to have undergone menopausal transition either from pre‐ or perimenopause to postmenopause (baseline *n* = 316). The *short*‐*term follow*‐*up* sample included 230 perimenopausal women who were followed until early postmenopause (mean follow‐up time 1.3 ± 0.7 years). The *long*‐*term follow*‐*up* sample included 148 women who were pre‐ or perimenopausal at ERMA baseline and postmenopausal at the time of the EsmiRs laboratory measurement (mean follow‐up time 3.9 ± 0.2 years). This sample also included a subgroup of women (*n* = 62) who were measured at both the short‐ and long‐term follow‐up points.

The baseline characteristics and body composition are described in Tables S1 and S2. Participants were a representative sample of Finnish 47–55‐year‐old women. At baseline, premenopausal women were about five months younger, had higher E2 and lower FSH, were more likely to use progestogenic contraception (either intra‐uterine device (IUD) or mini pills) or to have gone through hysterectomy, and had a lower diet quality score compared with the perimenopausal group (Supplemental Table 1). Although at the group mean level participants were slightly overweight (BMI 25.5 ± 3.9), half of them fulfilled normal weight criteria. For body composition variables, the proportional metrics of total fat mass (FM), total fat‐%, gynoid FM, right leg FM, gluteofemoral FM, and gluteofemoral fat‐% were lower in the premenopausal than the perimenopausal women (Table S2).

**TABLE 1:** Participant characteristics and adiposity variables during short‐ and long‐term follow‐ups

	Short‐term follow‐up			Long‐term follow‐up		
	Baseline	Follow‐up	*Change from baseline*	Baseline	Follow‐up	*Change from baseline*
**Background characteristics**	*n* = 230			*n* = 148		
E_2_, nmol/L	0.34 ± 0.27	0.24 ± 0.18	*−0.10 ± 0.33* ***	0.48 ± 0.50	0.20 ± 0.21	*−0.28 ± 0.54* ***
FSH, IU/L	36.1 ± 21.7	66.7 ± 28.1	*30.5 ± 31.7* ***	24.1 ± 21.8	80.4 ± 32.0	*56.3 ± 36.4* ***
Body mass, kg^a^	69.7 ± 11.1	70.3 ± 11.5	*0.7 ± 2.7* ***	69.8 ± 10.8	72.0 ± 12.1	*2.3 ± 3.6* ***
BMI, kg/m^2^ ^a^	25.6 ± 3.9	25.8 ± 4.1	*0.3 ± 1.0* ***	25.5 ± 3.9	26.3 ± 4.4	*0.8 ± 1.3* ***
**Lifestyle habits**	*n* = 230			*n* = 148		
SR‐PA, MET‐h/day^b^	4.46 ± 3.98	4.69 ± 3.68	*0.23 ± 3.00*	5.27 ± 4.56	4.86 ± 3.81	*−0.42 ± 3.72*
ACC‐PA, min/day^c^	51.9 ± 29.6	49.8 ± 23.9	*−2.1 ± 24.7*	54.4 ± 32.6	48.4 ± 28.9	*−6.0 ± 28.2* *
Diet quality score^d^	6.01 ± 2.18	5.89 ± 2.16	*−0.12 ± 1.60*	5.86 ± 2.50	5.83 ± 2.29	*−0.03 ± 1.90*
**Use of external hormones**						
*None*	*67.8%*	*64.3%* ***		*67.6%*	*66.2%* ***	
*Estrogen*	*0%*	*0.9%*		*0%*	*2.7%*	
*Progestogen*	*32.2%*	*19.6%*		*32.4%*	*15.5%*	
*Estrogen +Progestogen*	*0%*	*15.2%*		*0%*	*15.5%*	
**Adipokines**	*n* = 110			*n* = 68		
Leptin, ng/ml	42.4 ± 30.5	50.4 ± 38.2	*8.0 ± 18.2* ***	40.8 ± 30.2	54.3 ± 39.6	*13.5 ± 23.2* ***
Adiponectin, ng/ml	16644 ± 6232	18475 ± 7730	*1831 ± 4285* ***	16510 ± 6908	19669 ± 8979	*3159 ± 6241* ***
Resistin, pg/ml	18842 ± 7958	17243 ± 7556	*−1599 ± 5723* **	20481 ± 9053	17946 ± 7575	*−2536 ± 7353* *
**Total and regional fat**	*n* = 219			*n* = 132		
Total fat mass, kg	25.7 ± 8.8	26.4 ± 9.0**	*0.8 ± 2.5* ***	24.4 ± 8.8	27.1 ± 9.6	*2.6 ± 2.8* ***
Total fat‐%	35.8 ± 7.8	36.6 ± 7.5**	*0.8 ± 2.4* ***	34.2 ± 8.0	36.7 ± 7.9	*2.5 ± 2.3* ***
Trunk fat mass, kg	13.1 ± 5.4	13.6 ± 5.7**	*0.5 ± 1.7* ***	12.5 ± 5.3	14.2 ± 6.0	*1.7 ± 2.0* ***
Gynoid fat mass, kg	5.0 ± 1.4	5.1 ± 1.4**	*0.1 ± 0.5* **	4.7 ± 1.4	5.0 ± 1.5	*0.3 ± 0.5* ***
Android fat mass, kg	2.3 ± 1.0	2.4 ± 1.0**	*0.1 ± 0.3* ***	2.2 ± 1.0	2.5 ± 1.1	*0.3 ± 0.4* ***
Right leg fat mass, kg	4.5 ± 1.5	4.6 ± 1.5**	*0.1 ± 0.5* **	4.2 ± 1.5	4.5 ± 1.6	*0.3 ± 0.5* ***
Gluteofemoral fat mass, kg	10.5 ± 3.5	10.9 ± 3.5	*0.3 ± 1.0* ***	10.0 ± 3.4	11.0 ± 3.8	*1.0 ± 1.2* ***
Gluteofemoral fat‐%	36.7 ± 6.8	37.7 ± 6.6	*0.9 ± 2.1* ***	35.1 ± 7.0	37.5 ± 6.7	*2.4 ± 2.2* ***
Android‐to‐gynoid ratio	0.45 ± 0.14	0.46 ± 0.14	*0.01 ± 0.04* ***	0.45 ± 0.15	0.50 ± 0.15	*0.05 ± 0.05* ***
**Mid‐thigh fat**	*n* = 76			*n* = 17		
Subcutaneous adipose tissue area, cm^2^ ^e^	64.2 ± 15.8	65.7 ± 17.0	*1.5 ± 3.8* **	65.0 ± 17.5	69.3 ± 18.7	*4.3 ± 4.1* **
Muscle compartment adipose tissue area, cm^2^	9.4 ± 3.0	9.6 ± 3.1	*0.1 ± 1.1*	8.1 ± 2.2	9.5 ± 1.4	*1.3 ± 1.9* *
Muscle density, HU	53.1 ± 3.7	53.4 ± 3.9	*0.3 ± 1.7*	53.7 ± 4.0	50.0 ± 3.1	*−3.7 ± 3.7* **

Note

Values are presented as mean ± *SD*.

Abbreviations: E2, estradiol; FSH, follicle‐stimulating hormone; BMI, body mass index; SR‐PA, self‐reported physical activity; MET, metabolic equivalent of task; ACC‐PA, accelerometer‐measured moderate‐to‐vigorous physical activity; HU, Hounsfield unit.

^a^ Data missing, *n* = 12 from long‐term‐follow‐up.

^b^ Data missing, *n* = 3 from short‐term and *n* = 7 from long‐term follow‐up.

^c^ Data missing, *n* = 61 from short‐term and *n* = 21 from long‐term follow‐up.

^d^ Data missing, *n* = 6 from short‐term and *n* = 9 from long‐term follow‐up.

^e^ Data missing, *n* = 1 from short‐term follow‐up.

*** *p* < 0.001

** *p* < 0.01

* *p* < 0.05.

### 2.2 Body adiposity increased during short‐ and long‐term follow‐ups over the menopause

Short‐ and long‐term follow‐up group characteristics are presented in Table 1. Body mass and BMI increased on average by 1% and 3% during both follow‐ups respectively (*p* < 0.001 for all). Of the lifestyle habits, accelerometer‐measured moderate‐to‐vigorous physical activity (ACC‐PA) decreased in the long‐term follow‐up and the use of external hormones increased, shifting from contraceptive use toward HT. In both follow‐ups, serum leptin and adiponectin increased and serum resistin concentrations decreased.

All total and regional fat variables measured with dual‐energy X‐ray absorptiometry (DXA) increased during the follow‐ups. The mean relative increments were 2%–4% in the short‐term (*p* < 0.01 for all) and 7%–14% in the long‐term follow‐ups (*p* < 0.001 for all). Computed tomography (CT) was used to assess mid‐thigh adipose tissue area. Subcutaneous adipose tissue area increased by 2% during the short‐term follow‐up and 7% in the long‐term follow‐up (*p* < 0.01 for both). Muscle compartment adipose tissue area increased by an average of 16% (*p* < 0.05) and muscle density decreased by 7% (*p* < 0.05) during the long‐term but not the short‐term follow‐up.

### 2.3 Subgroup analysis of participants with three measurement points

**FIGURE 2:**
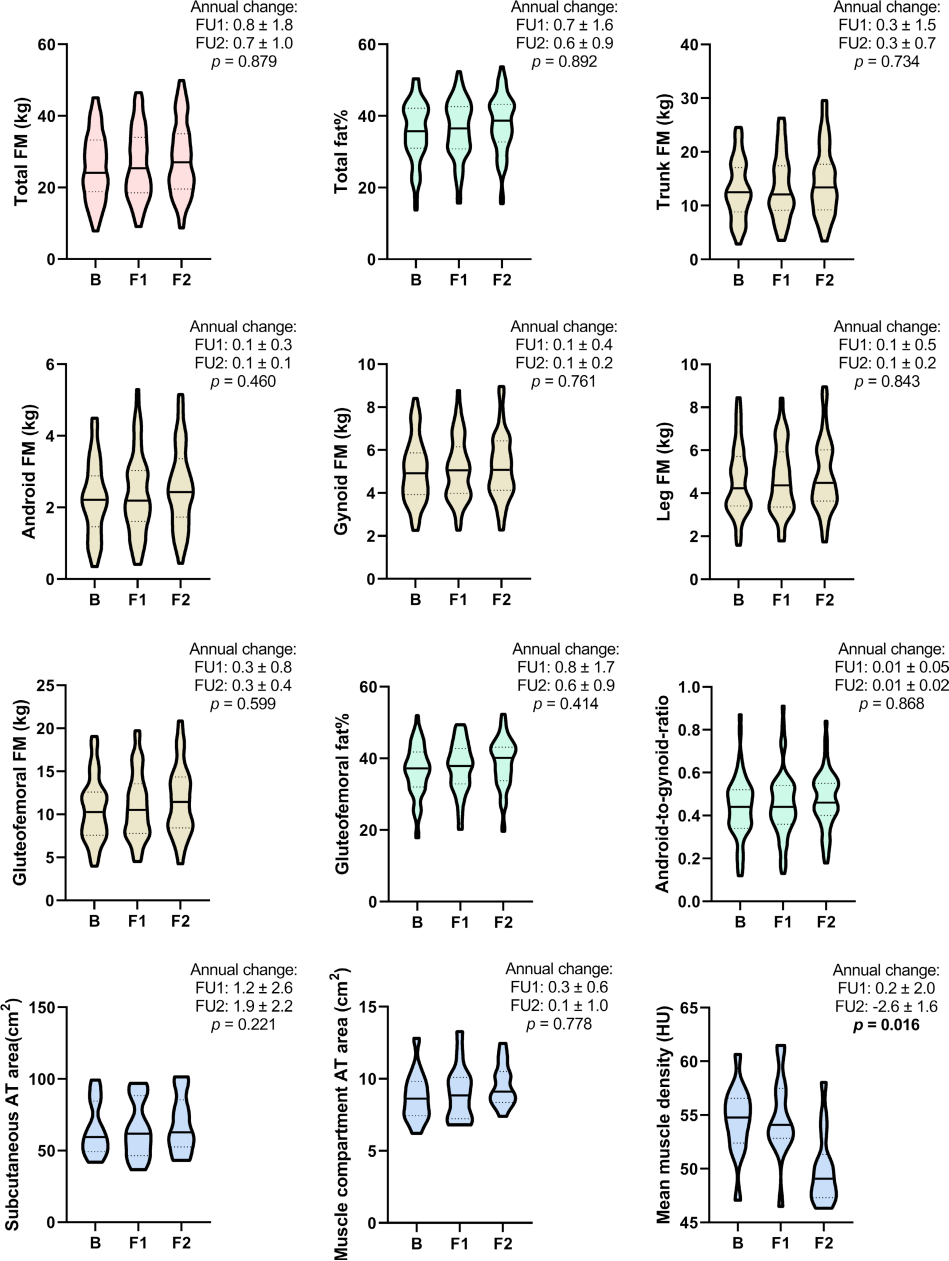
Body adiposity variables of participants assessed at three measurement points during the study (*n* = 59 for three upper rows and *n* = 14 for bottom row variables). Violin plots show the median (vertical line), interquartile ranges (dashed lines), and max‐ and min‐values (upper and lower limits). Annual changes are presented as median ± *SD*. B, baseline measurement; F1, first follow‐up measurement; F2, second follow‐up measurement; FU1, follow‐up from perimenopause to early postmenopause (time between B and F1); FU2, follow‐up from early postmenopause to later postmenopause (time between F1 and F2); FM, fat mass; AT, adipose tissue; HU, Hounsfield unit. Significant p‐values (*p* < 0.05) between annual changes are highlighted

Figure 2 presents adiposity characteristics for the women who were measured three times during the study (DXA, *n* = 59; CT, *n* = 14). In this subpopulation, the mean follow‐up time between baseline (B) and the first follow‐up point (F1) was 1.4 (0.4–3.6) years, and between F1 and the second follow‐up point (F2) 2.5 (0.7–3.7) years. Median annual changes in adipose tissue variables were calculated for both follow‐ups to compare the rate of change between the transition from perimenopause to early postmenopause (FU1, from B to F1) and progression through postmenopause thereafter (FU2, from F1 to F2). Only the rate of change in muscle density differed significantly between FU1 and FU2 (0.2 ± 2.0 HU/year vs −2.6 ± 1.6 HU/year, *p* = 0.016). Changes in sex hormones, body mass, BMI, physical activity, and adipokines were similar to the respective follow‐ups in the full sample (data not shown).

### 2.4 Progression of menopause and a healthier lifestyle are associated with adipose tissue accumulation in opposite ways

Linear mixed‐effect models were constructed to study associations between menopausal progression and total, regional and mid‐thigh fat variables, and to assess whether lifestyle habits (physical activity, diet quality score, and external hormone use) modulated these associations (Table 2). Self‐reported leisure‐time physical activity (SR‐PA) was chosen as the physical activity measure instead of ACC‐PA due to the larger number of valid measurements. Nonetheless, models using accelerometer data supported the following findings (data not shown). For all studied DXA fat variables, the progression of menopause was a significant predictor of adipose tissue accumulation (*p* < 0.001 for all). Higher diet quality score was associated with lower adiposity for all other variables (*p* < 0.05 for all) except for android‐to‐gynoid ratio. Higher physical activity was also associated with lower adiposity (*p* < 0.01 for all). Of the mid‐thigh fat variables, time was a significant predictor of subcutaneous adipose tissue area (*p* < 0.001). Higher physical activity was associated with lower subcutaneous adipose tissue area and higher muscle density (*p* < 0.05 for both).

**TABLE 2:** Linear mixed‐effect model results from DXA and CT variables with SR‐PA as a physical activity measure (total observations *n* = 620 for DXA and *n* = 165–167 for CT variables)

	Total fat mass (kg)			Trunk fat mass (kg)			Android fat mass (kg)			Gynoid fat mass (kg)			Right leg fat mass (kg)			Gluteofemoral fat mass (kg)			Android‐to‐gynoid ratio			Subcutaneous adipose tissue area (cm^2^)			Muscle compartment adipose tissue area (cm^2^)			Mean muscle density (HU)		
	B	95%	CI	B	95%	CI	B	95%	CI	B	95%	CI	B	95%	CI	B	95%	CI	B	95%	CI	B	95%	CI	B	95%	CI	B	95%	CI
*Univariable model*																														
Time	**0.69** ***	0.57	0.81	**0.46** ***	0.38	0.55	**0.09** ***	0.07	0.11	**0.08** ***	0.05	0.10	**0.07** ***	0.05	0.09	**0.27** ***	0.22	0.32	**0.01** ***	0.01	0.01	**1.24** ***	0.77	1.72	0.15	−0.11	0.30	**−0.46** **	−0.76	−0.17
*Multivariable model* ^a^																														
Time	**0.68** ***	0.55	0.80	**0.45** ***	0.37	0.54	**0.09** ***	0.07	0.11	**0.07** ***	0.05	0.09	**0.07** ***	0.04	0.09	**0.27** ***	0.21	0.32	**0.01** ***	0.01	0.01	**1.05** ***	0.49	1.60	−0.14	−0.03	0.32	−0.23	−0.58	0.12
Hormone use																														
E+P4	−4×10^−3^	−0.75	0.74	−0.07	−0.59	0.45	−0.05	−0.16	0.05	0.08	−0.05	0.22	2×10^−3^	−0.15	0.15	−0.07	−0.39	0.24	−0.01	−0.03	2×10^−3^	−0.08	−2.37	2.22	−7×10^−3^	−0.80	0.78	−0.86	−2.33	0.62
E	−0.12	−2.34	2.09	0.02	−1.51	1.55	−0.03	−0.34	0.27	−0.12	−0.52	0.29	−0.06	−0.49	0.36	−0.17	−1.10	0.76	0.01	−0.03	0.05	‐	‐	‐	‐	‐	‐	‐	‐	‐
P4	−0.19	−0.90	0.52	−0.24	−0.72	0.24	−0.02	−0.12	0.07	−0.01	−0.14	0.12	−0.06	−0.20	0.08	−0.18	−0.47	0.12	1×10^−4^	−0.01	0.01	0.37	−6.15	6.90	0.15	−2.17	2.47	−1.02	−5.61	3.58
DQS	**−0.23** **	−0.37	−0.09	**−0.16** **	−0.26	−0.07	**−0.02** **	−0.04	−0.01	**−0.04** **	−0.06	−0.01	**−0.03** *	−0.06	−0.01	**−0.08** **	−0.14	−0.02	−1×10^−3^	−4×10^−3^	1×10^−3^	−0.19	−0.66	0.27	−2×10^−3^	−0.15	0.15	0.05	−0.20	0.30
SR‐PA	**−0.17** ***	−0.25	−0.08	**−0.11** ***	−0.17	−0.06	**−0.02** ***	−0.03	−0.01	**−0.03** ***	−0.04	−0.01	**−0.03** **	−0.04	−0.01	**−0.07** ***	−0.10	−0.03	−3×10^−3**^	−4×10^−3^	−1×10^−3^	**−0.37** *	−0.69	−0.04	−0.05	−0.16	0.06	**0.25** *	0.04	0.46
Time x SR‐PA	−3×10^−4^	−0.04	0.04	4×10^−4^	−0.02	0.02	1×10^−3^	−4×10^−3^	0.01	3×10^−3^	−3×10^−3^	0.01	2×10^−3^	−5×10^−3^	0.01	−2×10^−3^	−0.02	0.01	3×10^−4^	−3×10^−4^	1×10^−3^	−0.18	−0.37	0.02	3×10^−3^	−0.06	0.07	0.10	−0.03	0.22

Abbreviations: DXA, dual‐energy X‐ray absorptiometry; CT, computed tomography; SR‐PA, centered self‐reported physical activity in MET‐hours/day; Note: DXA, dual‐energy X‐ray absorptiometry; CT, computed tomography; SR‐PA, centered self‐reported physical activity in MET‐hours/day; MET, metabolic equivalent of a task; HU, Hounsfield unit; B, unstandardized regression coefficient; CI, confidence interval; Time: follow‐up time in years; Hormone use: reference group is non‐hormone users; E, estrogen; P4, progestogen; DQS: centered diet quality score.

^a^ Model adjusted with centered baseline age and education level. Significant (*p* < 0.05) B values in bold.

*** *p* < 0.001

** *p* < 0.01

* *p* < 0.05.

### 2.5 Adipokines are associated with menopausal transition, and adiponectin and leptin are concomitantly associated with total and regional fat mass variables

**FIGURE 3:**
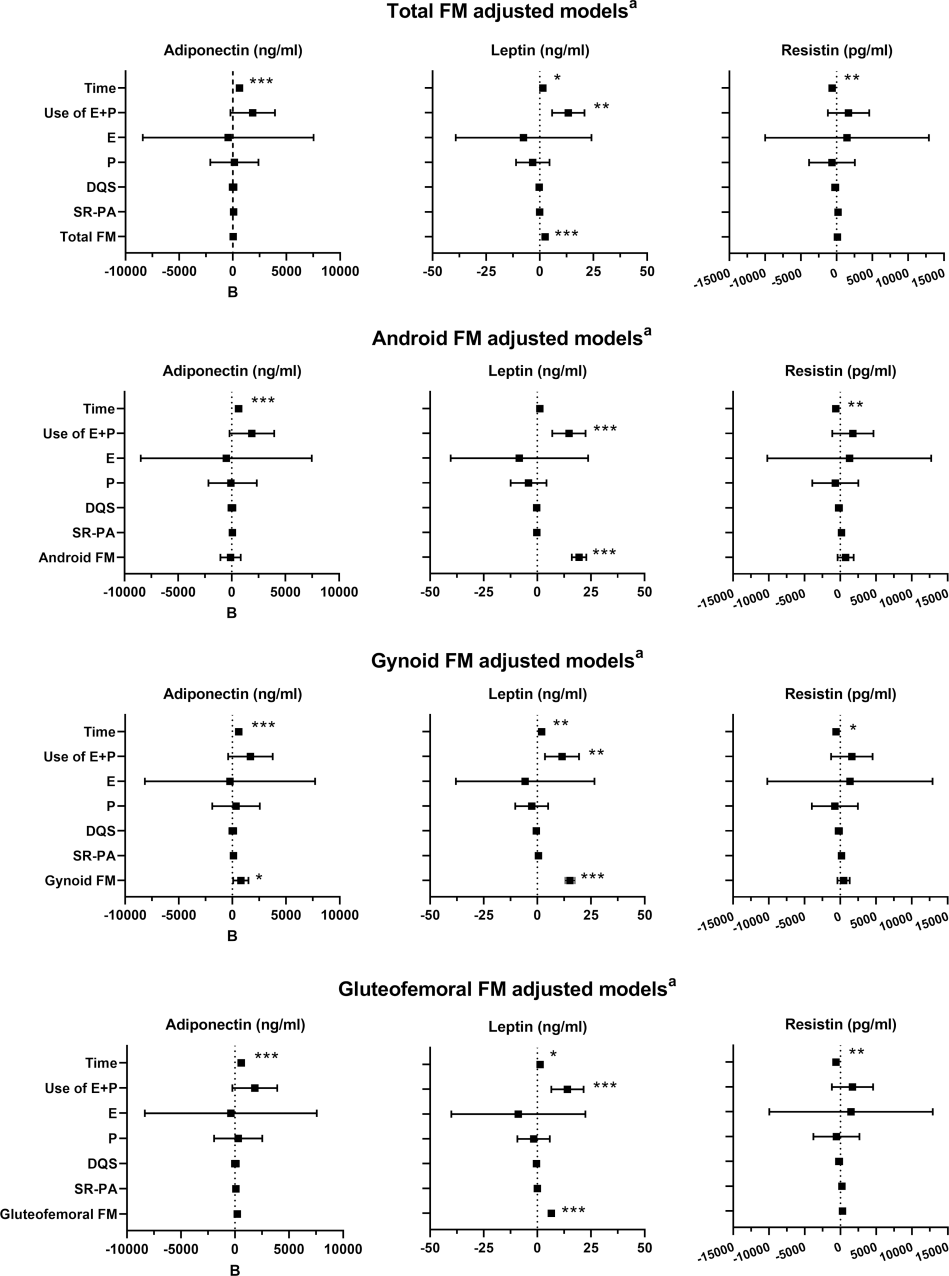
Results from the adjusted adipokine and adiposity variables linear mixed‐effect models (total observations *n* = 276). Black squares represent the B coefficient of the model covariates, while lines represent the 95% confidence interval. Time, follow‐up time in years; Hormone use, the reference group is non‐users; E, estrogen; P, progestogen; DQS, diet quality score; SR‐PA, self‐reported physical activity (MET‐hours/day); MET, metabolic equivalent of task; FM, fat mass (kg). All models were also adjusted for centered baseline age and education. ***p < 0.001 **p < 0.01, *p < 0.05

To study the associations between adipokines and selected adiposity variables, linear mixed‐effect models were constructed (Figure 3). Adiponectin levels were positively associated with menopause progression, even after adjusting the models for body adiposity (*p* < 0.001 for all). Of the FM variables, adiponectin was positively associated with gynoid FM (*p* < 0.05). Leptin was positively associated with menopause in the total, gynoid, and gluteofemoral FM‐adjusted models (*p* < 0.05 for all). Combined estrogen and progestogen use and the total and regional FMs of android, gynoid, and gluteofemoral area were positively associated with leptin levels (*p* < 0.01 for all). Resistin concentration was negatively associated with menopause in all adiposity models (*p* < 0.05 for all) but was not associated with any FM variables.

### 2.6 Fiber types differ in their lipid droplet content and oxidative and glycolytic capacities, but menopause is not associated with changes in these parameters

**FIGURE 4:**
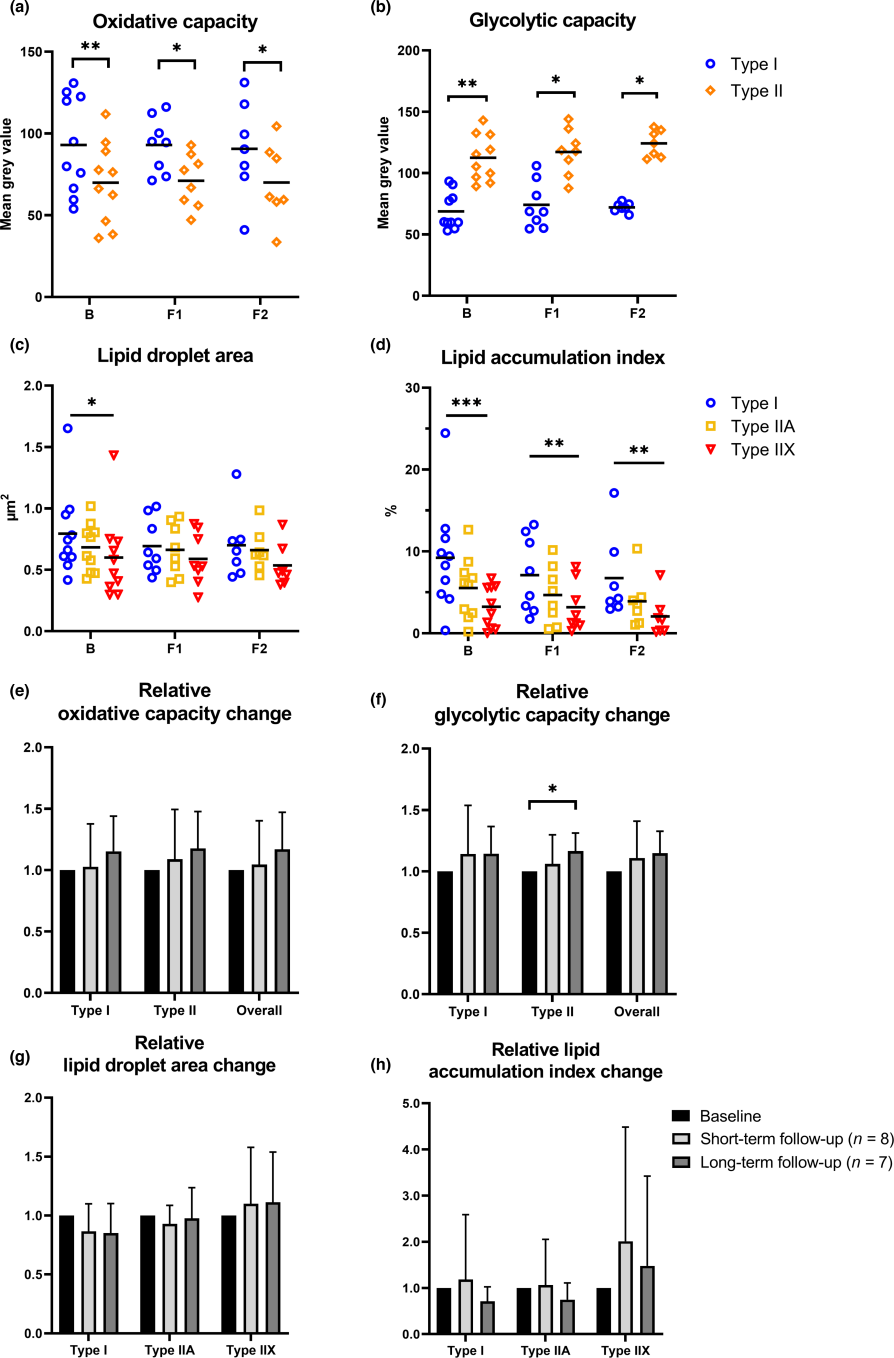
Biopsy results (*n* = 7–10). Oxidative and glycolytic capacities, lipid droplet area, and lipid accumulation index in different fiber types at baseline (B), short‐term follow‐up point (F1), and long‐term follow‐up point (F2) (Figure 4a‐d). In 4A‐D, horizontal lines represent mean values. Figure 4e‐h shows relative paired changes in oxidative and glycolytic capacity, lipid droplet area, and lipid accumulation index per fiber type, where baseline values were set as a reference value (=1) between short‐ and long‐term follow‐ups. Here, values are presented as mean ± *SD*. ***p < 0.001, **p < 0.01, *p < 0.05.

Muscle fiber oxidative capacity was investigated with succinate dehydrogenase and glycolytic capacity with α‐glycerophosphate dehydrogenase staining. Lipid accumulation was quantified with lipid droplet staining. First, oxidative and glycolytic capacities were studied between fiber types and separately at the B, F1, and F2 time points (Figure 4a,b **)**. Samples from 10 participants were analyzed at baseline, eight at F1, and seven at F2. None of the participants used HT. At all time points, type I fibers had higher oxidative capacity than type II fibers, whereas glycolytic capacity was higher in type II than in type I fibers (*p* < 0.05 for all). Secondly, single lipid droplet area and lipid accumulation index were studied separately among fiber types at all three time points (Figure 4c,d). At baseline, lipid droplet area was the largest in type I fibers (*p* < 0.05). No difference was observed at other time points. At all time points, lipid accumulation index was the largest in type I fibers (*p* < 0.01 for all).

Thirdly, to study the change in metabolic capacity and lipid accumulation during follow‐ups, baseline results were used as a reference value and paired relative changes between timepoints were investigated (*n* = 8 for the short‐term and *n* = 7 for the long‐term follow‐up) (Figure 4e‐h). Only glycolytic capacity in type II fibers in the long‐term follow‐up increased significantly (1 vs 1.16, *p* = 0.043, Figure 4f). Overall tissue oxidative and glycolytic capacities were calculated using the information from fiber type distribution as the overall metabolic capacity of a tissue depends on the ratio of different fiber types. During the follow‐ups, no significant changes were observed in the relative overall tissue oxidative or glycolytic capacity (Figure 4e,f), or in lipid droplet area or accumulation index (Figure 4g,h).

**FIGURE 5:**
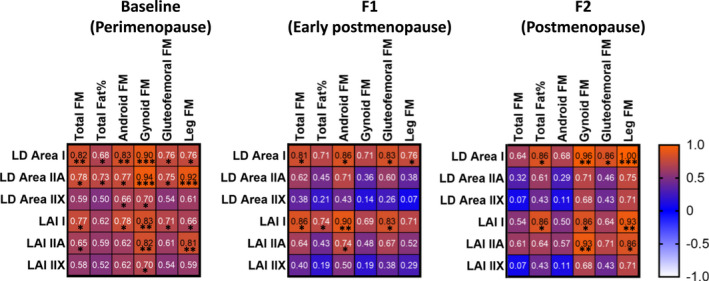
Correlation matrix of muscle fiber and body adiposity variables at three measurement points (*n* = 7–10). Correlation strength and direction are illustrated by correlation coefficient and background color. Red indicates a strong positive correlation and white indicates a strong negative correlation. F1, short‐term follow‐up point; F2, long‐term follow‐up point; FM, fat mass; LD, lipid droplet; LAI, lipid accumulation index. ***p < 0.001, **p < 0.01, *p < 0.05.

We also investigated associations between myofiber lipid and body adiposity variables at all measurement points (Figure 5). In type I fibers, lipid droplet area correlated positively with total FM, fat‐%, android FM, gynoid FM, gluteofemoral FM, and leg FM in at least two time points (*r_s_* =0.76–1.00, *p* < 0.05 for all). In type I fibers, lipid accumulation index correlated positively with all studied FM variables in at least two time points (*r_s_* =0.66–0.93, *p* < 0.05 for all). In type IIA fibers, lipid accumulation index correlated positively with gynoid and leg FM at baseline and F2 (*r_s_* =0.81–0.93, *p* < 0.05). For oxidative and glycolytic capacities, correlations were only found at single time points (results not shown). At F1, leg FM correlated positively with the glycolytic capacity of type II cells (*r_s_* =0.76, *p* = 0.037) and total tissue glycolytic capacity (*r_s_* =0.81, *p* = 0.022). At F2, oxidative capacity in type II cells and at the total tissue level correlated negatively with lipid accumulation index in type IIA cells (type II: *r_s_* = −0.71, *p* = 0.034; overall tissue *r_s_* = −0.82, *p* = 0.034).

## 3 DISCUSSION

In this longitudinal study over the menopausal transition, changes were observed in multiple adiposity measures, from circulating adipokines to regional body fat. Our results show that menopause contributes to body fat accumulation and is uniquely associated with systemic leptin, adiponectin, and resistin levels. We observed fat accumulation at multiple adipose depots from the total body to the limb level. The increase was detectable already during the short‐term follow‐up, which focused on the time between perimenopause and early postmenopause, and was more noticeable when the follow‐up time was extended to cover the transition from premenopause to further postmenopause. The increase in android FM was relatively large compared with total body or other regional depots. Leptin and adiponectin levels increased during both follow‐ups, while resistin levels decreased. At the cellular level, we observed muscle fiber type‐specific oxidative and glycolytic capacities and lipid accumulation index, but no changes during menopausal transition except for an increment in type II fiber glycolytic capacity. Lipid droplet area and lipid accumulation index, especially in type I fibers, were positively correlated with total and regional body adiposity measurements during menopause.

During the reproductive years, women typically have higher total body FM than men, especially in the gluteofemoral region due to hormonal and enzymatic actions (Wells, 2007). Increasing evidence from longitudinal studies suggests that the loss of female sex hormones during menopause shifts the site of adipose tissue accumulation toward the waist and android area (Lee et al., 2009; Lovejoy et al., 2008). This observation is supported by our data—although adiposity increased in all body regions, android‐to‐gynoid ratio also increased, indicating higher fat accumulation in the central body regions. Furthermore, we observed higher relative fat accumulation in upper than in lower body regions. For instance, during the long‐term follow‐up, FM increased by 14% in the trunk and android areas compared with increments of 6% and 10% in the gynoid and gluteofemoral areas respectively. The link between menopause and total body fatness is still inconclusive. The main contributor to increased total body fat has been suggested to be either menopause (Greendale et al., 2019; Lovejoy et al., 2008) or aging (Ambikairajah et al., 2019). In a longitudinal study of women aged 40–66, Guo et al. observed a 0.41 kg mean annual increase in body fat and found that postmenopausal women had more total body fat than similar aged pre‐ or perimenopausal women (Guo et al., 1999). We observed a mean annual increase of 0.68 kg of body fat over a maximum period of four years around menopause. This, and similar results from other groups (Greendale et al., 2019; Lovejoy et al., 2008), suggests that the increase in adiposity is accelerated around menopause, and that the increase is pronounced in white women (Marlatt et al., 2020). Menopausal changes are part of normal female aging, thus differentiating them in an observational study is difficult, yet it seems that menopause could have an accelerating effect on adiposity.

Physical activity is a widely studied approach to manage body weight and adiposity in all age groups, including middle‐aged women (Grindler & Santoro, 2015). In our study, we investigated associations between self‐reported as well as device‐measured physical activity level and body adiposity during menopausal transition. Similar to previous studies (Sternfeld et al., 2005), we also found that higher physical activity level was associated with lower body adiposity. Although associations between physical activity and body composition have been broadly studied in menopausal women, most studies are cross‐sectional or conducted in postmenopausal women. To the best of our knowledge, only one study in addition to ours has reported longitudinal associations between voluntary physical activity level and changes in body adiposity during menopause (Sternfeld et al., 2004). Although the participants in our study were relatively active (approx. 50 min of MVPA per day), this was insufficient to prevent fat accumulation during menopause, as also reported by Sternfeld et al. (2004). We also found that diet quality was significantly associated with several adiposity variables. Although we did not directly assess energy intake, the diet quality score has been shown to negatively correlate with the intake of total and saturated fat and sucrose (Masip et al., 2019), which might imply an overall higher use of calorie‐rich foods. Thus, these findings emphasize the importance of healthy dietary patterns for the management of body adiposity during middle age.

Adipokines play a vital role in tissue–tissue crosstalk and are broadly associated with different metabolic health parameters, such as total FM and insulin sensitivity. Our results suggest that regional differences in adipokine production, and especially gynoid area FM, have an important effect on adipokine levels (Figure 3). As menopause has previously been shown to be associated with declines in metabolic health parameters (Hyvärinen et al., 2021; Wang et al., 2018), and adiponectin as an indicator of good metabolic health (Recinella et al., 2020), the observed increase in adiponectin levels was unexpected and seems counterintuitive, but has also been reported by others (Lee et al., 2009). As the participants in our cohort had fairly healthy lifestyles, it is possible that they were more protected from metabolic deterioration than might have been expected based on the observed changes in body composition (Table 1). Longitudinal associations between leptin and menopause progression have previously been investigated in a few studies (Lee et al., 2009; Sowers et al., 2008), which suggested that body adiposity is the main contributor to leptin levels. We found that leptin levels were positively associated with several body adiposity variables, but also with the menopausal transition (Figure 3). We also observed that the use of estrogen‐ and progestogen‐containing menopausal HT was associated with higher leptin levels, which contradicts our results on menopausal progression. E2 level has previously been shown to correlate with leptin levels in younger women (Geber et al., 2012), but not in women during menopause (Springer et al., 2014). As leptin is a manifold contributor to total body metabolism, more research is clearly needed to clarify its associations with female sex hormones. Resistin, the third adipokine we investigated, has been previously linked to increased BMI and body adiposity in some (Recinella et al., 2020; Zhang et al., 2015), but not all studies (Chu et al., 2006). In cross‐sectional studies, resistin levels do not differ according to menopausal state (Gupta et al., 2008; Hong et al., 2007), while a longitudinal study by Sowers et al. found a decrease in resistin levels after menopause (Sowers et al., 2008). Here, we also observed a negative association with menopausal progression when the models were adjusted for FM variables. Mechanisms between E2 and resistin have been studied in animal models (Caja & Puerta, 2007), but the results remain inconclusive. As resistin is a potential candidate in preventing type 2 diabetes (Su et al., 2019), which is an emerging risk for postmenopausal women, more longitudinal studies are warranted.

Cellular changes in skeletal muscle adiposity during menopause are understudied, and to the best of our knowledge, our study is the first to address this issue. Intracellular lipid droplets are dynamic in nature, but their concentration seems to correlate with total body adiposity (He et al., 2001). Similar to previous studies (He et al., 2001), we also found that lipid accumulation differs between the three muscle fiber types, but we did not detect a change in lipid droplet size or accumulation index during the follow‐ups (Figure 4). As we did observe a decrease in muscle density in the long‐term follow‐up CT scans, it is possible that the accumulated low‐density tissue is either located between the muscle fibers or is not adipose tissue, but connective tissue instead (Edmunds et al., 2018). We also observed strong positive correlations, especially between lower limb DXA and *m*. *vastus lateralis* biopsy adiposity variables, suggesting that the correlations between cell and tissue adiposity may be region‐specific. Our results on the metabolic enzyme potential of skeletal muscle during menopause are also the first of their kind. Although we observed a small increase in glycolytic capacity of type II fibers, we cannot exclude the possible confounding effect of varying freezing times between the longitudinal samples. Nonetheless, the clear differences observed in oxidative and glycolytic capacities between different muscle fiber types might play a role in individual metabolic maintenance in middle age. As skeletal muscle is a metabolically important tissue with a high rate of energy consumption, more research in menopausal women is warranted.

The current study has several strengths and limitations. The strengths include careful characterization of menopausal status and a comprehensive set of adiposity measurements supplemented with measures of systemic adipokines and cellular muscle adiposity analysis. We used a modified version of the STRAW+10 criteria (Harlow et al., 2012), which is widely considered the gold standard for characterizing menopausal stages. Body composition was also measured using gold standard methods, DXA and CT. Limitations of the study include that due to exclusion criteria, participants represented a cohort of relatively healthy middle‐aged Caucasian women, hindering their generalizability to other populations. Unfortunately, we were not able to separate visceral adipose fat from the other adipose depots of the central body. Thus, our analysis of android adiposity includes both subcutaneous and visceral depots. The small number of available muscle biopsy samples also prevents us from drawing stronger conclusions about cellular modifications.

We conclude that menopausal transition is associated with increasing regional and total body adiposity, particularly in central body regions. Physical activity alone does not mitigate increased adiposity, but higher physical activity and diet quality are associated with lower adiposity at baseline, and may therefore delay the accumulation of adipose tissue. Menopausal transition correlates with adipokine release, reflecting associations between female sex hormones and adipose tissue, yet the implications remain unclear. At the cellular level, skeletal muscle fiber characteristics are not affected by the menopausal transition. Information from the current study can be used in health education of adult women to emphasize the importance of physical activity and a healthy diet in maintaining beneficial body composition.

## 4 EXPERIMENTAL PROCEDURES

### 4.1 Study design and participants

This study used longitudinal data from ERMA (Estrogenic Regulation of Muscle Apoptosis) and EsmiRs (The Estrogen, MicroRNAs, and the Risk of Metabolic Dysfunction) studies (Figure 1). In total, 1393 women were assigned to baseline menopausal groups based on their current FSH levels and menstrual bleeding diaries. Participants with conditions affecting normal ovarian function or the use of estrogen‐containing medication were excluded. Assigned groups were premenopausal, early perimenopausal, late perimenopausal, and postmenopausal, based on the modified STRAW+10 guidelines (Kovanen et al., 2018). A subgroup of the perimenopausal women was invited to take part in the *ERMA longitudinal study*, in which women were followed individually to early postmenopause. During the follow‐up, participants visited the laboratory every three to six months based on their previous FSH levels and bleeding diaries. When the participant was categorized as early postmenopausal, the baseline measurements were repeated. These data form the “*short*‐*term follow*‐*up*.” After starting the follow‐up, three participants were re‐categorized as premenopausal, but as they reached early postmenopause during short‐term follow‐up, they were included in the current study.

Four years after the ERMA baseline, 206 women took part in the EsmiRs laboratory measurements, where the baseline measurements were repeated. Each participant's menopausal status was again determined based on FSH level, menstrual bleeding data, and additional information from the ERMA longitudinal study for those who participated in it. Participants categorized as postmenopausal were included in the “*long*‐*term follow*‐*up*.”

Baseline ERMA data were collected during 2015–2016 and the ERMA follow‐up measurements were performed between August 2015 and early 2019. EsmiRs data collection started in January 2019 and finished in March 2020.

The study followed the Declaration of Helsinki. All participants provided written informed consent, and the study was approved by the ethical committee of the Central Finland Health Care District (ERMA 8 U/2014 and EsmiRs 9 U/2018).

### 4.2 Hormone measurements

Fasting serum samples were taken from the antecubital vein between 7 and 10 AM. At baseline, women with a regular menstrual cycle were asked to visit the laboratory between cycle days 1 to 5. Serum E2, FSH, sulfated dehydroepiandrosterone (DHEAS), and sex‐hormone‐binding globulin (SHBG) were measured with IMMULITE 2000 XPi (Siemens Healthcare Diagnostics, UK). Serum leptin, adiponectin, and resistin levels were measured with a Quansys Multiplex‐kit (custom kits HCUM190820‐ID and HA2M200303‐ID, Quansys Biosciences, Utah, USA).

After baseline measurements, participants started the follow‐ups. During the short‐term follow‐up, elevated FSH levels were verified with control blood samples. A participant was only categorized as early postmenopausal after two elevated FSH samples accompanied by at least six months without menstruation. The participant was then invited to the final short‐term follow‐up visit for physiological measurements, during which the E2 and FSH levels were again measured. For the final short‐term follow‐up analysis, the E2 and FSH values from the last regular follow‐up visit and final follow‐up visits were averaged to minimize the effect of daily fluctuation. Hormone levels from the participants who started using hormone therapy during the short follow‐up were only collected from the final follow‐up visit. For participants in the long‐term follow‐up, baseline hormone measurements were repeated during the EsmiRs laboratory visit. Serum leptin, adiponectin, and resistin levels were also measured at baseline.

### 4.3 Fat mass measurements

Measurements were done after overnight fasting. Total, trunk, gynoid, android, and right leg FM and total fat‐% were analyzed from the DXA scans (LUNAR Prodigy; GE Healthcare, Chicago, IL). Android‐to‐gynoid ratio was calculated by dividing android FM (kg) by gynoid FM (kg). Gluteofemoral area was outlined manually using the iliac crest line as the upper limit and the knee joint as the lower limit of a rectangle (Peppa et al., 2013). Corresponding FM and fat‐% were calculated from images. DXA measurement of right leg FM was chosen to accompany the quantitative CT scans that were also taken from the right mid‐thigh.

The right mid‐thigh was scanned at the level of the muscle biopsy with CT (Siemens Somatom Emotion scanner, Siemens, Erlangen, Germany). Subcutaneous and muscle compartment (area inside muscle fascia) adipose tissue area was measured using appropriate thresholds in Python Software (version 3.6). From the cross‐sectional image, the muscle portion including the femur was first separated using a U‐net machine learning algorithm or manually if needed. Adipose tissue area was separated from muscle and bone tissues using Hounsfield Unit (HU) limits, and mean muscle density was calculated. Images were analyzed using ImageJ Software (v.1.52, NIH, USA) and Python.

### 4.4 Muscle biopsies

Muscle biopsies were collected from the middle of *m*. *vastus lateralis* using suction‐modified Bergström needle biopsies under local anesthesia. All visible connective and adipose tissues were removed, and the sample was divided into three parts. Two parts were assigned to biochemical and molecular biology analyses and were snap‐frozen in liquid nitrogen. The third part assigned for histology was embedded transversely on a cork with TissueTek and frozen in isopentane cooled in liquid nitrogen. All samples were stored at −150°C until analysis.

### 4.5 Immunohistochemistry and lipid droplet staining

Transverse, 10‐µm sections were cut with a cryostat at −21°C. Slides were air‐dried and fixed in 4% paraformaldehyde, incubated with 100 mM glycine, and blocked in 5% goat serum (GS). Primary and secondary antibodies were added to 5% GS. Myosin heavy chain distribution was analyzed with antibodies against type I (A4.951, 2 µg/µl, Developmental Studies Hybridoma Bank (DSHB), University of Iowa, IA, USA) and type IIX (6H1, 5 µg/µl, DSHB). Anti‐laminin (L9393, 1:250, Sigma‐Aldrich, St. Louis, MO, USA) was used to detect cell borders. Primary antibodies were incubated at +4°C o/n, and the attachment was visualized with fluorescent secondary antibodies Alexa Fluor 405 goat anti‐mouse (A31553, 1:500, Invitrogen, Oregon, USA), Alexa Fluor 647 goat anti‐mouse (A21238, 1:500, Invitrogen), and Alexa Fluor 488 goat anti‐rabbit (A11034, 1:500; Invitrogen). LD540 (0.1µg/ml, produced in the University of Jyväskylä Chemistry department) was incubated in sections for 20 min RT to stain neutral lipids. After rinsing with PBS, sections were mounted with Mowiol‐Dabco and imaged with confocal microscopy (LSM 700, Axio Observer, Zeiss, Oberkochen, Germany). Laminin signal was enhanced, and broken cells were excluded in ImageJ with the Trainable Weka Segmentation plugin and later by human inspection. Cell segmentation and the measurement of lipid droplet size, number, and area fraction were achieved as previously reported (Fachada et al., 2022). Lipid accumulation index was calculated as the combined area of lipid droplets divided by mean fiber size (Goodpaster et al., 2000).

### 4.6 Enzyme histology

Serial 12‐µm transverse sections were cut at −21°C. Enzyme activities of succinate dehydrogenase (SDH) and α‐glycerophosphate dehydrogenase (GPD) were measured with histological staining. SDH incubation medium consisted of 1 mg/ml NBT (N‐6876, Sigma) and 27 mg/ml sodium succinate (S‐2378, Sigma) in 0.2 M phosphate buffer, pH 7.4. Sections were incubated in prewarmed solution for 90 min at 37.2°C, washed with H_2_O, and mounted with Mowiol. GPD medium consisted of 1.2 mM NBT, 2.3 mM menadione (47775, Sigma), and 9.3 mM alfa‐glycerophosphate (G6501, Sigma) in 0.05 M Tris‐Buffer, pH 7.4. Sections were incubated in prewarmed solution for 40 min at 37.2°C, washed with H_2_O, and cleared with acetone. Lastly, sections were rinsed with H_2_O and mounted with Mowiol. Samples were imaged with Olympus BX50 (10×/0.30) (Olympus, Tokyo, Japan). In ImageJ (v. 1.53c), images were transformed into 8‐bit grayscale format. At least 55 cells per sample were manually cropped, and mean gray value was calculated, where 0 indicated black and 255 indicated white. To describe staining intensity more intuitively, mean cell gray values were subtracted from the maximum gray value of 255 and the resulting values were used for further analysis. Enzyme staining was paired with myosin fiber type staining in serial sections. 12‐µm sections were air‐dried and blocked in 10% GS. Primary and secondary antibodies were added to 10% GS. Fiber types were analyzed with antibodies against type I (A4.951, 2 µg/µl, DSHB) and type II (A4.74, 2 µg/µl, DSHB) fibers. Anti‐laminin (L9393, 1:250, Sigma‐Aldrich) was used to detect cell borders. Sections were incubated in primary antibodies at RT for 1 h, washed, and incubated with secondary antibodies Alexa Fluor 546 goat anti‐mouse (A11003, 1:500, Invitrogen) and Alexa Fluor 488 goat anti‐rabbit (A11034, 1:500, Invitrogen) at RT for 1 h. Sections were mounted with Mowiol‐Dabco and imaged with confocal microscopy. Images were analyzed with ImageJ. Corresponding fibers were localized manually between histological and immunohistological images.

### 4.7 Physical activity

Physical activity was evaluated with a structured questionnaire (Kujala et al., 1998) and hip‐worn accelerometer as reported previously (Laakkonen et al., 2017). Briefly, the questionnaire included four questions about the frequency, intensity, and duration of leisure‐time physical activity bouts and the average time spent in active commuting. Based on the answers, metabolic equivalent of a task (MET) hours per day for leisure‐time physical activity was calculated. Objective physical activity was assessed with an accelerometer worn for seven consecutive days (ActiGraph GT3X+ or wGT3X+, Pensacola, FL, USA). The data analysis process has been reported previously (Hyvärinen et al., 2019). Briefly, the amount of moderate‐to‐vigorous physical activity (MVPA) was assessed using triaxial vector magnitude cutoff point of 2690 counts per minute and the daily averages were adjusted to 16 h of daily wear time (Laakkonen et al., 2017).

### 4.8 Diet quality

Diet was assessed using food‐frequency questionnaires and quantified using the diet quality score as done previously (Juppi et al., 2020). Shortly, diet quality score was calculated based on 11 components that are characteristic of a healthy diet, as described in the Nordic Nutrition Recommendations 2012. Regular intake of foods such as vegetables, fruits and berries, dark bread, low‐fat dairy, fish and nuts, and seeds was considered beneficial. Moreover, limited intake of processed meats, processed grain products, sugar‐sweetened beverages, fast food, and sweet or salty snacks was also favored. Each component was worth 1 point, and the maximum score available was 11 points. A higher score reflected a healthier diet.

### 4.9 Health status and medications

Information about medical conditions and prescription medicine that may have affected adiposity was collected from the questionnaires throughout the follow‐ups. Participants diagnosed with cancer during the study were excluded. The suitability of non‐insulin‐treated type 2 diabetics (*n* = 2) and new thyroid medication users (*n* = 7) for analysis was investigated, but since sensitivity analysis revealed no major difference in the results, they were included in the analysis.

The use of exogenous sex hormones was determined from questionnaires, resulting in a four‐class variable: non‐user, only estrogen‐user, only progestogen‐user, and combined estrogen and progestogen‐user. Transdermal (patches, gels, and sprays), oral (tablets), and intra‐uterine preparations were included, but local intravaginal estrogen therapy was not. The use of estrogen and progestogen preparations during menopause may affect blood FSH levels due to a negative feedback loop mechanism. We conducted sensitivity analyses (data not shown) between menopausal stages and external hormone use, and found that pre‐ and perimenopausal progestogen users had lower FSH levels compared with non‐users. At postmenopause, estrogen use was associated with higher E2 levels and decreased FSH levels when compared to non‐estrogen users. Yet, the difference was not consistent in all comparisons. At postmenopause, progestogen use was not associated with altered hormone levels.

### 4.10 Background variables

Anthropometric measurements were done after overnight fasting. Body mass was measured with a digital scale and height with a stadiometer. Body mass index (BMI) was calculated as body mass divided by height squared (kg/m^2^). Data about smoking (current/quitter/never smoked), alcohol consumption (portions per week), and level of education (primary, secondary, and tertiary) was determined with a questionnaire. Baseline missing information for education was *n* = 14 (*n* = 6 in premenopausal and *n* = 8 in perimenopausal group). These data were completed using information from the follow‐up questionnaires, except for one completely missing questionnaire in the perimenopausal group.

### 4.11 Statistical analysis

All variables were evaluated for normality and parametric tests were used whenever possible. Independent samples *t*‐test, chi‐squared test, and Mann–Whitney *U*‐tests were used to compare baseline characteristics between pre‐ and perimenopausal groups. Paired *t*‐test, Wilcoxon signed‐rank test, and marginal homogeneity tests were used to test for differences in baseline characteristics, adipokines, and adiposity variables between baseline and follow‐ups. Wilcoxon signed‐rank test was used to test for differences in annual changes within participants whose body composition was measured at three time points. Friedman test was used to compare nonparametric lipid droplet size and lipid accumulation index between three fiber types. Wilcoxon signed‐rank test was used to assess the difference in relative changes during follow‐up in cell variables. Due to non‐normal distributions, Spearman correlations were calculated and visualized with GraphPad Prism (v.9.1.1) to examine associations between cell and selected adipose tissue variables.

Linear mixed‐effect models were created to examine associations between the adiposity measurements and covariates during the follow‐ups. Since the models included information from both the short‐ and long‐term follow‐up and because menopausal status at baseline and four‐year follow‐up was not uniform, duration of follow‐up in years was selected to represent time. To investigate associations between time and each of the adipose tissue mass variables, models were controlled for education, baseline mean‐centered age, mean‐centered physical activity, mean‐centered diet quality score, the use of external hormones, and the interaction between time and physical activity (fixed effects). The models were constructed using unstructured longitudinal correlation matrix, and intercept and time were used as random effects. Longitudinal associations between adipokines and adiposity variables were investigated with similar linear mixed‐effect models, but without the interaction between time and physical activity. The possible effect of smoking was tested, but because we did not see associations in any of the variables, it was left out of the final models. All variables and covariates were evaluated for outliers. A value was considered to be an outlier if it was above 3rd quartile +3*interquartile range or 1st quartile −3*interquartile range. Based on the extreme values in body adiposity (*n* = 3), SR‐PA (*n* = 5), ACC‐PA (*n* = 3), and adipokines *(n* = 3), participants were removed from the analyses. Multicollinearity between covariates was assessed with variance inflation factor analyses. The model assumptions were tested using Q–Q plots of residuals. Statistical data analysis was carried out using IBM SPSS Statistics Software version 26 (Chicago, IL, USA), and a *p*‐value < 0.05 was considered statistically significant. Due to the observational nature of the study with predetermined associations of interest, the results are presented without multiple comparison corrections. With the large number of statistical comparisons in the study, this may increase the risk of type I error.

## 5 PERMISSION TO REPRODUCE MATERIAL FROM OTHER SOURCES

For graphical abstract, BioRender's Academic License has been granted.

## CONFLICT OF INTEREST

The authors have no conflict of interest to declare.

## AUTHORS’ CONTRIBUTIONS

VK, EKL, and SS designed the ERMA study and UK, and PA contributed to the planning. EKL designed the EsmiRs study, and VK and SS substantially contributed to the planning. UMK and PA also supported planning of the study. HKJ was responsible for the majority of the laboratory measurements and analysis including adipokine analysis, tissue staining, microscopy imaging, DXA, and CT analyses, while EKL supervised them. HKJ performed statistical analysis with the guidance of MH. SS performed the CT scanning and supervised the analyses. VF was responsible for preparing a script for confocal microscopy image analysis and performed the analysis. NC prepared and analyzed CT scans. PA and UMK offered clinical knowledge and guidance to data analysis. JEK prepared the diet quality score. HS was the clinician responsible for performing muscle biopsies. VK and EKL provided funding for the study. SK assisted with laboratory assays. HKJ prepared the first version of the manuscript. SS, VF, MH, NC, PA, JEK, HS, UMK, VK, SK, and EKL have participated in the interpretation of the results and critically commented on the manuscript during the writing process. All the authors have read and approved the final manuscript.

## Supporting information

Fig S1

Table S1‐S2

## Data Availability

The data that support the findings of this study are available on request from the project leader (eija.k.laakkonen@jyu.fi). The data are not publicly available due to privacy or ethical restrictions.

## References

[acel13621-bib-0001] Ambikairajah, A. , Walsh, E. , Tabatabaei‐Jafari, H. , & Cherbuin, N. (2019). Fat mass changes during menopause: A metaanalysis. American Journal of Obstetrics and Gynecology, 221(5), 393–409.e50. 10.1016/j.ajog.2019.04.023 31034807

[acel13621-bib-0002] Caja, S. , & Puerta, M. (2007). Control by reproduction‐related hormones of resistin expression and plasma concentration. Hormone and Metabolic Research, 39(7), 501–506. 10.1055/s-2007-982515 17611902

[acel13621-bib-0003] Chait, A. , & den Hartigh, L. J. (2020). Adipose tissue distribution, inflammation and its metabolic consequences, including diabetes and cardiovascular disease. Frontiers in Cardiovascular Medicine, 7(22). 10.3389/fcvm.2020.00022 PMC7052117 32158768

[acel13621-bib-0004] Chu, M. C. , Cosper, P. , Orio, F. , Carmina, E. , & Lobo, R. A. (2006). Insulin resistance in postmenopausal women with metabolic syndrome and the measurements of adiponectin, leptin, resistin, and ghrelin. American Journal of Obstetrics and Gynecology, 194(1), 100–104. 10.1016/j.ajog.2005.06.073 16389017

[acel13621-bib-0005] Edmunds, K. , Gíslason, M. , Sigurðsson, S. , Guðnason, V. , Harris, T. , Carraro, U. , & Gargiulo, P. (2018). Advanced quantitative methods in correlating sarcopenic muscle degeneration with lower extremity function biometrics and comorbidities. PLoS One, 13(3), e0193241. 10.1371/journal.pone.0193241 29513690 PMC5841751

[acel13621-bib-0046] Fachada, V. , Rahkila, P. , Fachada, N. , Turpeinen, T. , Kujala, U. M. & Kainulainen, H. (2022). Enlarged PLIN5‐uncoated lipid droplets in inner regions of skeletal muscle type II fibers associate with type 2 diabetes. Acta Histochemica, 124(3). 10.1016/j.acthis.2022.151869 35220055

[acel13621-bib-0006] Fasshauer, M. , & Blüher, M. (2015). Adipokines in health and disease. Trends in Pharmacological Sciences, 36(7), 461–470. 10.1016/j.tips.2015.04.014 26022934

[acel13621-bib-0007] Geber, S. , Brandão, A. H. F. , & Sampaio, M. (2012). Effects of estradiol and FSH on leptin levels in women with suppressed pituitary. Reproductive Biology and Endocrinology, 10(1), 45. 10.1186/1477-7827-10-45 22703959 PMC3495667

[acel13621-bib-0008] Goodpaster, B. H. , Kelley, D. E. , Thaete, F. L. , He, J. , & Ross, R. (2000). Skeletal muscle attenuation determined by computed tomography is associated with skeletal muscle lipid content. Journal of Applied Physiology, 89(1), 104–110. 10.1152/jappl.2000.89.1.104 10904041

[acel13621-bib-0009] Goossens, G. H. , Jocken, J. W. E. , & Blaak, E. E. (2021). Sexual dimorphism in cardiometabolic health: The role of adipose tissue, muscle and liver. Nature Reviews Endocrinology, 17(1), 47–66. 10.1038/s41574-020-00431-8 33173188

[acel13621-bib-0010] Greendale, G. A. , Sternfeld, B. , Huang, M. H. , Han, W. , Karvonen‐Gutierrez, C. , Ruppert, K. , Cauley, J. A. , Finkelstein, J. S. , Jiang, S.‐F. , & Karlamangla, A. S. (2019). Changes in body composition and weight during the menopause transition. JCI Insight, 4(5), e124865. 10.1172/jci.insight.124865 PMC6483504 30843880

[acel13621-bib-0011] Grindler, N. M. , & Santoro, N. F. (2015). Menopause and exercise. Menopause (New York, NY), 22(12), 1351–1358. 10.1097/gme.0000000000000536 26382311

[acel13621-bib-0012] Guo, S. S. , Zeller, C. , Chumlea, W. C. , & Siervogel, R. M. (1999). Aging, body composition, and lifestyle: The Fels Longitudinal Study. The American Journal of Clinical Nutrition, 70(3), 405–411. 10.1093/ajcn/70.3.405 10479203

[acel13621-bib-0013] Gupta, P. , Harte, A. , Sturdee, D. W. , Sharma, A. , Barnett, A. H. , Kumar, S. , & McTernan, P. G. (2008). Effects of menopausal status on circulating calcitonin gene‐related peptide and adipokines: Implications for insulin resistance and cardiovascular risks. Climacteric, 11(5), 364–372. 10.1080/13697130802378493 18781480

[acel13621-bib-0014] Harlow, S. D. , Gass, M. , Hall, J. E. , Lobo, R. , Maki, P. , Rebar, R. W. , Sherman, S. , Sluss, P. M. , & de Villiers, T. J. (2012). Executive summary of the stages of reproductive aging workshop +10: Addressing the unfinished agenda of staging reproductive aging. Menopause, 19(4), 387–395. 10.1097/gme.0b013e31824d8f40 22343510 PMC3340903

[acel13621-bib-0015] He, J. , Watkins, S. , & Kelley, D. E. (2001). Skeletal muscle lipid content and oxidative enzyme activity in relation to muscle fiber type in type 2 diabetes and obesity. Diabetes, 50(4), 817–823. 10.2337/diabetes.50.4.817 11289047

[acel13621-bib-0016] Hong, S. C. , Yoo, S. W. , Cho, G. J. , Kim, T. , Hur, J. Y. , Park, Y. K. , Lee, K. W. , & Kim, S. H. (2007). Correlation between estrogens and serum adipocytokines in premenopausal and postmenopausal women. Menopause, 14(5), 835–840. 10.1097/gme.0b013e31802cddca 17667144

[acel13621-bib-0017] Hyvärinen, M. , Juppi, H.‐K. , Taskinen, S. , Karppinen, J. E. , Karvinen, S. , Tammelin, T. H. , Kovanen, V. , Aukee, P. , Kujala, U. M. , Rantalainen, T. , Sipilä, S. , & Laakkonen, E. K. (2021). Metabolic health, menopause, and physical activity—A 4‐year follow‐up study. International Journal of Obesity, 46(3), 544–554. 10.1038/s41366-021-01022-x 34802032 PMC8605777

[acel13621-bib-0018] Hyvärinen, M. , Sipilä, S. , Kulmala, J. , Hakonen, H. , Tammelin, T. H. , Kujala, U. M. , Kovanen, V. , & Laakkonen, E. K. (2019). Validity and reliability of a single question for leisure‐time physical activity assessment in middle‐aged women. Journal of Aging and Physical Activity, 28(2), 231–241. 10.1123/japa.2019-0093 31585436

[acel13621-bib-0019] Juppi, H.‐K. , Sipilä, S. , Cronin, N. J. , Karvinen, S. , Karppinen, J. E. , Tammelin, T. H. , Aukee, P. , Kovanen, V. , Kujala, U. M. , & Laakkonen, E. K. (2020). Role of menopausal transition and physical activity in loss of lean and muscle mass: A follow‐up study in middle‐aged Finnish women. Journal of Clinical Medicine, 9(5), 1588. 10.3390/jcm9051588 PMC7290663 32456169

[acel13621-bib-0020] Kovanen, V. , Aukee, P. , Kokko, K. , Finni, T. , Tarkka, I. M. , Tammelin, T. , Kujala, U. M. , Sipilä, S. , & Laakkonen, E. K. (2018). Design and protocol of estrogenic regulation of muscle apoptosis (ERMA) study with 47 to 55‐year‐old women’s cohort: Novel results show menopause‐related differences in blood count. Menopause (New York, NY), 25(9), 1020–1032. 10.1097/GME.0000000000001117 PMC6110369 29738416

[acel13621-bib-0021] Kujala, U. M. , Kaprio, J. , Sarna, S. , & Koskenvuo, M. (1998). Relationship of leisure‐time physical activity and mortality: The Finnish twin cohort. JAMA, 279(6), 440–444. 10.1001/jama.279.6.440 9466636

[acel13621-bib-0022] Laakkonen, E. K. , Kulmala, J. , Aukee, P. , Hakonen, H. , Kujala, U. M. , Lowe, D. A. , Kovanen, V. , Tammelin, T. , & Sipilä, S. (2017). Female reproductive factors are associated with objectively measured physical activity in middle‐aged women. PLoS One, 12(2), e0172054. 10.1371/journal.pone.0172054 28225786 PMC5321412

[acel13621-bib-0023] Lee, C. G. , Carr, M. C. , Murdoch, S. J. , Mitchell, E. , Woods, N. F. , Wener, M. H. , Chandler, W. L. , Boyko, E. J. , & Brunzell, J. D. (2009). Adipokines, inflammation, and visceral adiposity across the menopausal transition: A prospective study. The Journal of Clinical Endocrinology and Metabolism, 94(4), 1104–1110. 10.1210/jc.2008-0701 19126626 PMC2682462

[acel13621-bib-0024] Liu, X. , Chan, H. C. , Ding, G. , Cai, J. , Song, Y. , Wang, T. , & Huang, H. (2015). FSH regulates fat accumulation and redistribution in aging through the Gαi/Ca 2+/CREB pathway. Aging Cell, 14(3), 409–420. 10.1111/acel.12331 25754247 PMC4406670

[acel13621-bib-0025] Lovejoy, J. C. , Champagne, C. M. , de Jonge, L. , Xie, H. , & Smith, S. R. (2008). Increased visceral fat and decreased energy expenditure during the menopausal transition. International Journal of Obesity, 32(6), 949–958. 10.1038/ijo.2008.25 18332882 PMC2748330

[acel13621-bib-0026] Manolopoulos, K. N. , Karpe, F. , & Frayn, K. N. (2010). Gluteofemoral body fat as a determinant of metabolic health. International Journal of Obesity, 34(6), 949–959. 10.1038/ijo.2009.286 20065965

[acel13621-bib-0027] Marlatt, K. L. , Redman, L. M. , Beyl, R. A. , Smith, S. R. , Champagne, C. M. , Yi, F. , & Lovejoy, J. C. (2020). Racial differences in body composition and cardiometabolic risk during the menopause transition: A prospective, observational cohort study. American Journal of Obstetrics and Gynecology, 222(4), 365.e1–365.e18. 10.1016/j.ajog.2019.09.051 31610152 PMC7141969

[acel13621-bib-0028] Masip, G. , Keski‐Rahkonen, A. , Pietiläinen, K. H. , Kujala, U. M. , Rottensteiner, M. , Väisänen, K. , Kaprio, J. , & Bogl, L. H. (2019). Development of a food‐based diet quality score from a short FFQ and associations with obesity measures, eating styles and nutrient intakes in Finnish twins. Nutrients, 11(11). 10.3390/nu11112561 PMC6893528 31652865

[acel13621-bib-0029] Newell‐Fugate, A. E. (2017). The role of sex steroids in white adipose tissue adipocyte function. Reproduction (Cambridge, England), 153(4), R133–R149. 10.1530/REP-16-0417 28115579

[acel13621-bib-0031] Papadakis, G. E. , Hans, D. , Rodriguez, E. G. , Vollenweider, P. , Waeber, G. , Marques‐Vidal, P. , & Lamy, O. (2018). Menopausal hormone therapy is associated with reduced total and visceral adiposity: The osteolaus cohort. The Journal of Clinical Endocrinology and Metabolism, 103(5), 1948–1957. 10.1210/jc.2017-02449 29596606

[acel13621-bib-0032] Peppa, M. , Koliaki, C. , Papaefstathiou, A. , Garoflos, E. , Katsilambros, N. , Raptis, S. A. , Hadjidakis, D. I. , & Dimitriadis, G. D. (2013). Body composition determinants of metabolic phenotypes of obesity in nonobese and obese postmenopausal women. Obesity, 21(9), 1807–1814. 10.1002/oby.20227 23696298

[acel13621-bib-0033] Recinella, L. , Orlando, G. , Ferrante, C. , Chiavaroli, A. , Brunetti, L. , & Leone, S. (2020). Adipokines: New potential therapeutic target for obesity and metabolic, rheumatic, and cardiovascular diseases. Frontiers in Physiology, 11, 1431. 10.3389/fphys.2020.578966 PMC7662468 33192583

[acel13621-bib-0034] Schiaffino, S. , & Reggiani, C. (2011). Fiber types in mammalian skeletal muscles. Physiological Reviews, 91(4), 1447–1531. 10.1152/physrev.00031.2010 22013216

[acel13621-bib-0035] Sowers, M. R. , Wildman, R. P. , Mancuso, P. , Eyvazzadeh, A. D. , Karvonen‐Gutierrez, C. A. , Rillamas‐Sun, E. , & Jannausch, M. L. (2008). Change in adipocytokines and ghrelin with menopause. Maturitas, 59(2), 149–157. 10.1016/j.maturitas.2007.12.006 18280066 PMC2311418

[acel13621-bib-0036] Springer, A. M. , Foster‐Schubert, K. , Morton, G. J. , & Schur, E. A. (2014). Is there evidence that estrogen therapy promotes weight maintenance via effects on leptin? Menopause (New York, NY), 21(4), 424–432. 10.1097/GME.0000000000000117 PMC3968225 24149922

[acel13621-bib-0037] Sternfeld, B. , Bhat, A. K. , Wang, H. , Sharp, T. , & Quesenberry, C. P. (2005). Menopause, physical activity, and body composition/fat distribution in midlife women. Medicine and Science in Sports and Exercise, 37(7), 1195–1202. 10.1249/01.mss.0000170083.41186.b1 16015138

[acel13621-bib-0038] Sternfeld, B. , Wang, H. , Quesenberry, C. P. Jr , Abrams, B. , Everson‐Rose, S. A. , Greendale, G. A. , & Sowers, M. (2004). Physical activity and changes in weight and waist circumference in midlife women: Findings from the study of women’s health across the nation. American Journal of Epidemiology, 160(9), 912–922. 10.1093/aje/kwh299 15496544

[acel13621-bib-0039] Straight, C. R. , Voigt, T. B. , Jala, A. V. , Chase, J. D. , Ringham, O. R. , Ades, P. A. , Toth, M. J. , & Miller, M. S. (2019). Quadriceps lipid content has sex‐specific associations with whole‐muscle, cellular, and molecular contractile function in older adults. The Journals of Gerontology: Series A, 74(12), 1879–1886. 10.1093/gerona/gly235 PMC6853688 30428006

[acel13621-bib-0040] Su, K.‐Z. , Li, Y.‐R. , Zhang, D. I. , Yuan, J.‐H. , Zhang, C.‐S. , Liu, Y. , Song, L.‐M. , Lin, Q. , Li, M.‐W. , & Dong, J. (2019). Relation of circulating resistin to insulin resistance in type 2 diabetes and obesity: A systematic review and meta‐analysis. Frontiers in Physiology, 10, 1399. 10.3389/fphys.2019.01399 31803062 PMC6877503

[acel13621-bib-0041] Tanner, C. J. , Barakat, H. A. , Dohm, G. L. , Pories, W. J. , MacDonald, K. G. , Cunningham, P. R. G. , Swanson, M. S. , & Houmard, J. A. (2002). Muscle fiber type is associated with obesity and weight loss. American Journal of Physiology‐Endocrinology and Metabolism, 282(6), 1191. 10.1152/ajpendo.00416.2001 12006347

[acel13621-bib-0042] Wang, H. , & Eckel, R. H. (2009). Lipoprotein lipase: From gene to obesity. American Journal of Physiology‐Endocrinology and Metabolism, 297(2), 271–288. 10.1152/ajpendo.90920.2008 19318514

[acel13621-bib-0043] Wang, Q. , Ferreira, D. L. S. , Nelson, S. M. , Sattar, N. , Ala‐Korpela, M. , & Lawlor, D. A. (2018). Metabolic characterization of menopause: Cross‐sectional and longitudinal evidence. BMC Medicine, 16(1), 17. 10.1186/s12916-018-1008-8 29402284 PMC5800033

[acel13621-bib-0044] Wells, J. C. K. (2007). Sexual dimorphism of body composition. Best Practice & Research Clinical Endocrinology & Metabolism, 21(3), 415–430. 10.1016/j.beem.2007.04.007 17875489

[acel13621-bib-0045] Zhang, J. , Pronyuk, K. H. O. , Kuliesh, O. V. & Chenghe, S. (2015). Adiponectin, resistin and leptin: Possible markers of metabolic syndrome. Endocrinology & Metabolic Syndrome, 4(4). 10.4172/2161-1017.1000212

