# Supplementary material for: Total and regional body adiposity increases during menopause—evidence from a follow‐up study
Source: Aging Cell. 2022 May 4;21(6):e13621. doi: 10.1111/acel.13621 (PMC9197413; doi:10.1111/acel.13621)
Supplement: Supplementary file 1 — Fig S1 [file ACEL-21-e13621-s001.pdf]

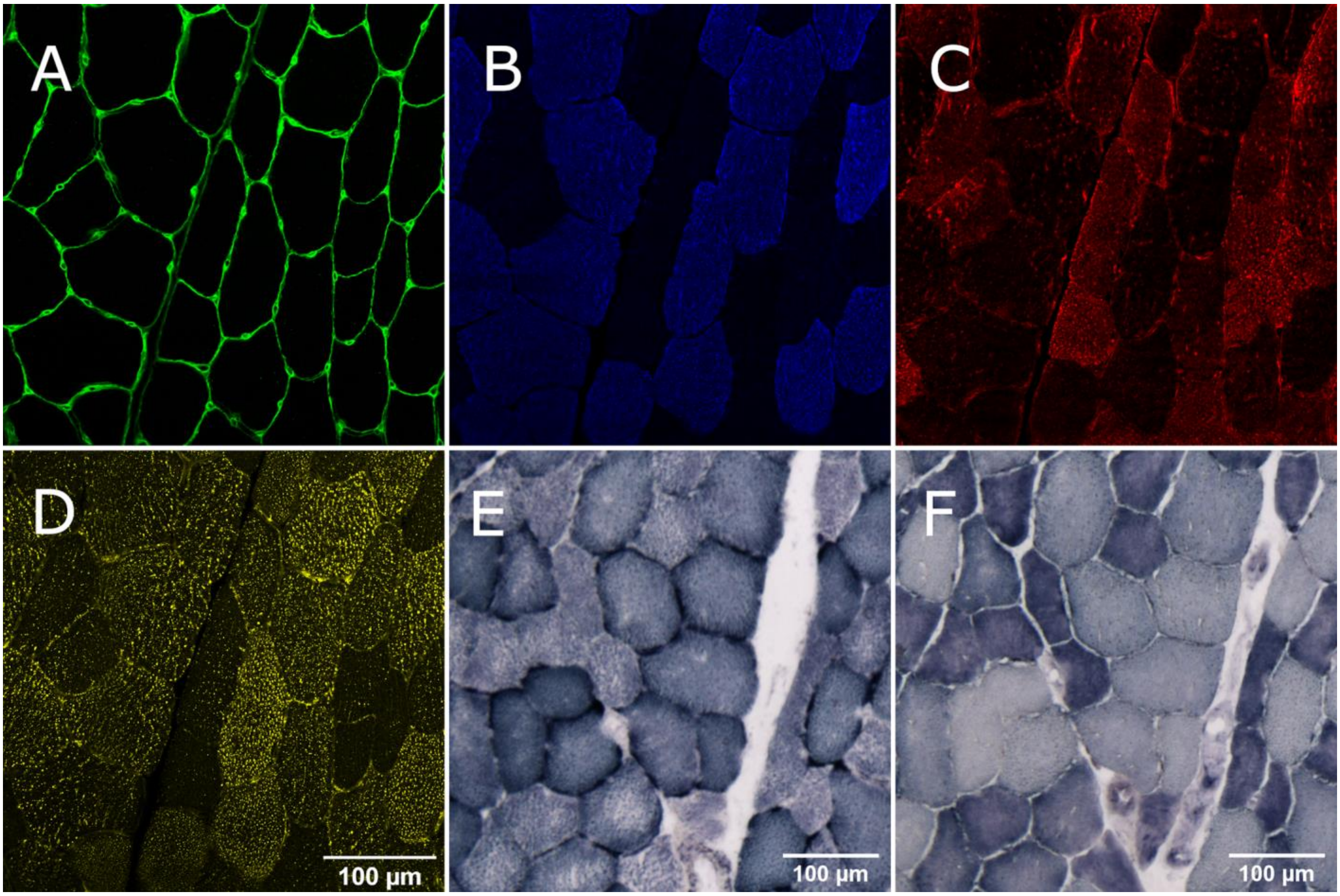

Supplemental Figure 1. Representative staining images. A: Cell borders, B: Type I fibers, C: Type IIX fibers, D: Lipid droplets, E: Succinate dehydrogenase, F: α-glycerophosphate dehydrogenase. Images A-D are layers of a single confocal microscopy image (sections B and C enhanced here only for visual purposes). Images E and F are from sequential sections.
