# Supplementary material for: Total and regional body adiposity increases during menopause—evidence from a follow‐up study
Source: Aging Cell. 2022 May 4;21(6):e13621. doi: 10.1111/acel.13621 (PMC9197413; doi:10.1111/acel.13621)
Supplement: Supplementary file 2 — Table S1‐S2 [file ACEL-21-e13621-s002.pdf]

## SUPPLEMENTARY MATERIAL

**Supplemental Table 1**. Baseline characteristics of the participants taking part either in the short-term, long-term or both follow-ups.

Baseline population consists of the following subpopulations:

- 54 participants, who were premenopausal at ERMA baseline and were re-measured only at long-term follow-up.
- 230 participants, who were perimenopausal at ERMA baseline and completed the short-term follow-up.
- 32 participants, who were perimenopausal at ERMA baseline and were re-measured only at long-term follow-up.

| | **All (*n* = 316)** | **Premenopausal (*n* = 54)** | **Perimenopausal (*n* = 262)** |
|---|---|---|---|
| **Age and systemic hormones** | | | |
| Age, y | 51.4 ± 1.9 | 51.0 ± 1.7 | 51.5 ± 1.9* |
| $E_2$, nmol/L | 0.40 ± 0.40 | 0.70 ± 0.71 | 0.33 ± 0.27*** |
| FSH, IU/L | 30.2 ± 22.2 | 7.9 ± 3.7 | 34.8 ± 21.6*** |
| DHEAS, µmol/l [a] | 2.73 ± 1.40 | 2.49 ± 1.48 | 2.77 ± 1.38 |
| SHBG, nmol/l [a] | 56.4 ± 24.3 | 60.7 ± 32.0 | 55.5 ± 22.4 |
| **Demographics** | | | |
| Education level [a] | | | |
| *Primary* | *2.2%* | *0.0%* | *2.7%* |
| *Secondary* | *51.7%* | *55.6%* | *51.0%* |
| *Tertiary* | *46.0%* | *44.4%* | *46.4%* |
| Bleeding status | | | |
| *Natural* | *59.5%* | *35.2%* | *64.5%**** |
| *IUD* | *33.9%* | *50.0%* | *30.5%* |
| *Hysterectomy* | *6.6%* | *14.8%* | *5.0%* |
| **Lifestyle habits** | | | |
| SR-PA, MET-h/day [b] | 4.67 ± 4.07 | 4.79 ± 3.80 | 4.64 ± 4.12 |
| ACC-PA, min/day [c] | 53.6 ± 30.3 | 53.5 ± 27.1 | 53.6 ± 31.0 |
| Diet quality score [d] | 5.84 ± 2.32 | 5.27 ± 2.72 | 5.95 ± 2.22* |
| Smoking | | | |
| *Never* | *69.6%* | *74.1%* | *68.7%* |
| *Quitter* | *23.7%* | *20.4%* | *24.4%* |
| *Current smoker* | *6.6%* | *5.6%* | *6.9%* |
| Alcohol use, units/week [e] | 4.0 ± 3.6 | 3.7 ± 3.1 | 4.1 ± 3.7 |

Values are presented as mean ± SD. E2, estradiol; FSH, follicle-stimulating hormone; DHEAS, dehydroepiandrosterone sulfate; SHBG, sex hormone binding globulin; IUD, intrauterine device (includes also other progestogenic contraception preparations); SR-PA, self-reported physical activity; MET, metabolic equivalent of a task; ACC-PA, accelerometer-measured moderate-to-vigorous physical activity. [a] Data missing, $n = 1$ from perimenopausal group. [b] Data missing, $n = 3$ from premenopausal and $n = 5$ from perimenopausal group. [c] Data missing, $n = 8$ from premenopausal and $n = 48$ from perimenopausal group. [d] Data missing, $n = 3$ from premenopausal and $n = 7$ from perimenopausal group. [e] Data missing, $n = 2$ from premenopausal and $n = 5$ from perimenopausal group. ***$p < 0.001$, **$p < 0.01$, *$p < 0.05$ between menopausal groups.

**Supplemental Table 2**. Baseline anthropometrics and body composition results of the participants taking part either in the short-term, long-term or both follow-ups.

Not all participants took part in all of the anthropometrics and body composition measurements both at baseline and follow-up visit. The baseline population with a minimum of scale measured body mass consists of the following subpopulations:

- 49 participants, who were premenopausal at ERMA baseline and were re-measured only at long-term follow-up.
- 230 participants, who were perimenopausal at ERMA baseline and completed the short-term follow-up.
- 28 participants, who were perimenopausal at ERMA baseline and were re-measured only at long-term follow-up.

| | **All (*n* = 307)** | **Premenopausal (*n* = 49)** | **Perimenopausal (*n* = 258)** |
|---|---|---|---|
| Body mass, kg | 69.6 ± 10.9 | 68.7 ± 10.4 | 69.7 ± 11.0 |
| Height, cm | 165.3 ± 5.5 | 166.2 ± 5.1 | 165.1 ± 5.6 |
| BMI, kg/m$^2$ | 25.5 ± 3.9 | 24.9 ± 3.6 | 25.6 ± 3.9 |
| *Underweight (<18.5)* | *0%* | *0%* | *0%* |
| *Normal weight (18.5–24.99)* | *51.8%* | *57.1%* | *50.8%* |
| *Overweight (25.0–29.99)* | *34.5%* | *34.7%* | *34.5%* |
| *Obese (>30)* | *13.7%* | *8.2%* | *14.7%* |
| **Total and regional fat** [a] | | | |
| Total fat mass, kg | 25.1 ± 8.7 | 22.8 ± 8.0 | 25.5 ± 8.8* |
| Total fat-% | 35.1 ± 7.8 | 32.4 ± 7.6 | 35.6 ± 7.8** |
| Trunk fat mass, kg | 12.8 ± 5.3 | 11.7 ± 4.8 | 13.0 ± 5.4 |
| Gynoid fat mass, kg | 4.9 ± 1.4 | 4.4 ± 1.4 | 5.0 ± 1.4* |
| Android fat mass, kg | 2.2 ± 1.0 | 2.0 ± 0.9 | 2.3 ± 1.0 |
| Right leg fat mass, kg | 4.4 ± 1.5 | 3.9 ± 1.5 | 4.5 ± 1.5* |
| Gluteofemoral fat mass, kg | 10.1 ± 3.4 | 9.1 ± 3.0 | 10.3 ± 3.4* |
| Gluteofemoral fat-% | 36.0 ± 6.9 | 33.5 ± 6.8 | 36.4 ± 6.8** |
| Android-to-gynoid-ratio | 0.45 ± 0.14 | 0.45 ± 0.16 | 0.45 ± 0.14 |

Values are presented as mean ± SD. BMI, body mass index. [a] Data missing $n = 1$ from premenopausal group and $n = 9$ from perimenopausal group. ***$p < 0.001$, **$p < 0.01$, *$p < 0.05$ between menopausal groups
